# Influence of Resistance Training on Neuromuscular Function and Physical Capacity in ALS Patients

**DOI:** 10.1155/2017/1436519

**Published:** 2017-05-17

**Authors:** L. Jensen, J. B. Djurtoft, R. D. Bech, J. L. Nielsen, L. H. Jørgensen, H. D. Schrøder, U. Frandsen, P. Aagaard, L. G. Hvid

**Affiliations:** ^1^Department of Sports Science and Clinical Biomechanics, SDU Muscle Research Cluster, University of Southern Denmark, 5230 Odense M, Denmark; ^2^Institute of Clinical Research, Clinical Pathology, SDU Muscle Research Cluster, University of Southern Denmark, 5000 Odense C, Denmark; ^3^Institute of Clinical Research, The Orthopaedic Research Unit, University of Southern Denmark, 5000 Odense C, Denmark

## Abstract

**Objectives:**

The present study aimed to explore the effect of resistance training in patients with amyotrophic lateral sclerosis (ALS), a disease characterized by progressive motor neuron loss and muscle weakness.

**Materials and Methods:**

Following a 12-week “lead-in” control period, a population of ALS patients from Funen, Denmark, completed a 12-week resistance training program consisting of 2-3 sessions/week. Neuromuscular function (strength and power) and voluntary muscle activation (superimposed twitch technique) were evaluated before and after both control and training periods. Physical capacity tests (chair rise and timed up and go), the revised ALS functional rating scale (ALSFRS-R) scores, and muscle cross sectional area (histology) were also assessed.

**Results:**

Of twelve ALS patients assessed for eligibility, six were included and five completed the study. Training did not significantly affect the ALSFRS-R score, and loss of neuromuscular function (strength and power) increased following the training period. However, an improved functionality (chair rise) and an increase in greatly hypertrophied type II fibres combined with an increase in atrophied fibres following the training period compared to the control period were observed.

**Conclusion:**

In this small study, the present form of resistance training was unable to attenuate progressive loss of neuromuscular function in ALS, despite some changes in physical capacity and morphology.

## 1. Introduction

Amyotrophic lateral sclerosis (ALS) is a progressive neurodegenerative disease affecting motor neurons in the spinal cord, brain stem, and motor cortex, resulting in muscle weakness and atrophy and eventually death [1].

In young as well as old healthy individuals resistance training appears highly effective for reducing muscle weakness and improving neuromuscular function (i.e., inducing gains in skeletal muscle strength and power) [2–4]. Historically, exercise in ALS has been thought to exacerbate muscle weakness, increase fatigue, and accelerate disease progression [5]. However, recent studies in ALS mice and patients—although limited in number—have challenged this view. In addition to an early case report showing positive effects of resistance training in ALS patients [6], it was first reported that exercise could improve the clinical course (Spinal Norris scores) and the performance in respiratory functional tests following exercise [7, 8]. Very recently it was reported in a randomized controlled trial that 6 months combined resistance and aerobic training led to improvements in the revised ALSFRS (ALS functional rating scale) [9]. Other observations in small cohorts include improved ALSFRS scores, quality of life, and maximal muscle strength measured by the Quantitative Muscle Assessment system following three months combined resistance type and flexibility training [10] and improved ALSFRS and decreased muscle spasticity following three months resistance type training [11].

Spinal motor neuron death results in partial denervation of skeletal muscle and is accompanied by sprouting and reinnervation, which in turn increase the size of the remaining motor units (MUs) [12]. The loss of MUs has been reported to half in each 6-month period during the first year in ALS, hereafter diminishing more slowly [13]. In addition, remaining MUs seem less efficient and fatigue more easily in ALS patients compared to healthy age-matched controls [14]. While this loss of MUs may likely have a negative effect on neuromuscular function, exercise may be able to diminish the loss of MUs and thus partly preserve neuromuscular function. Another important aspect of neuromuscular function relate to voluntary muscle activation, which yields a percentage measure of the neural input that reaches a given muscle [4].

It is still debated whether resistance training promotes or prevents progression of motor neuron degeneration in ALS. With this exploratory study we wanted to add to the existing knowledge by applying evaluation methods typically used in classical exercise science including measures of neuromuscular function (e.g., muscle strength) and voluntary muscle activation, which all remain unknown in ALS.

Therefore, the aim of this study was to explore the effect of resistance training on the ALSFRS-R as well as by evaluating voluntary muscle activation, neuromuscular function, histology, and functionality in ALS patients, as these evaluation methods could potentially serve as valuable endpoints when conducting larger training studies.

## 2. Methods

### 2.1. Study Design

The study is an open-label trial using a repeated measures design consisting of a 12-week “lead-in period” to benchmark individual disease progression (control period) followed by a 12-week resistance training intervention (training period). Information on the study design and data describing histopathological changes due to natural disease progression during the control period have previously been published [15].

Test and training was performed at the Department of Sports Science and Clinical Biomechanics at the University of Southern Denmark and Odense University Hospital. The control period served as a basis for estimating individual disease progression assuming a variable, yet linear decline in motor function over the 24-week study period, as this has previously been found to be a good approximation [16, 17]. We acknowledge that this is an assumption, but for the exploratory purposes of the study and the novel application of evaluation methods, we consider it reasonable.

Assessment of physical capacity, neuromuscular function, voluntary muscle activation, and skeletal muscle biopsies were collected at baseline (BL, *T* = 0 wks) and before (Pre, *T* = 12 wks) and after (Post, *T* = 24 wks) the training intervention. Data were obtained from the leg that was weakest at baseline. Participants were familiarized with all tests prior to performing baseline measures to minimize learning effects.

Participants were informed of risks associated with the study and provided written, informed consent before inclusion. The study was performed according to the Declaration of Helsinki and was approved by the local ethics committee of the Region of Southern Denmark (S-20100116). The trial was registered at clinicaltrials.gov (ID: NCT01504009).

### 2.2. Subjects

Six patients (aged 62.5 ± 8.8 years) diagnosed with amyotrophic lateral sclerosis (ALS) met the inclusion criteria and volunteered to participate in the study (Table 1). Inclusion criteria were diagnosis of definite or probable ALS based on the El Escorial criteria, ambulatory at onset of study, and being able to travel to training and test centre. Exclusions criteria were neurological or other serious medical problems and noncompliance with study protocol. At the time of inclusion all patients were able to walk unassisted. See [15] for further participant details.

### 2.3. Sample Size

The present study utilises patients sampled from one population (the island Funen, Denmark), and as such they are representative of the ALS population in general. The evaluation methods are not commonly used with ALS patients, which made it difficult to estimate the minimal clinically relevant effect and furthermore made it inapplicable to perform a power calculation prior to the study.

### 2.4. Resistance Training Intervention

The exercise protocol consisted of resistance exercise performed on nonconsecutive days 2-3 times per week. Exercises targeting both the upper and lower body (leg press, knee extension, leg curl, calf raises, lateral arm pull, seated rows, chest press, shoulder press, abdominal crunches, and back extension) were included in an alternating program utilising 6 exercises each session. Training was performed in small groups supervised by two experienced physical trainers and each session was initiated with a 5 min warm-up on a stationary bike (50–100 watts). The initial two weeks were used as familiarization with 3 sets of 12 repetitions at 15-repetition maximum (RM) load, and hereafter the training load progressed throughout the study concluding with 2 sets of 5 repetitions at 6 RM (by use of 5 RM testing). Continuous adjustments in exercises were made to ensure optimal compliance, as some exercises became difficult to perform due to individual disease progressions. Within 15 min after each training session protein supplementation was provided to all subjects (24 EN%, 18 g protein, Nutridrink, Nutricia, Denmark).

### 2.5. Functional Evaluation

Experienced health personnel evaluated functional ability by use of (i) the revised ALS functional rating scale (ALSFRS-R) questionnaire [18], (ii) 30 s chair rise (number of chair stands performed in 30 seconds) [19], and (iii) the timed up and go (TUG) [20].

### 2.6. Neuromuscular Function

For evaluation of maximal knee extensor strength, handgrip strength, and plantar flexor strength (elaborated below) subjects were instructed to contract as fast and forcefully as possible for 3-4 seconds during each trial. A minimum of 3 trials was performed for each interspersed by 1 min rest intervals. Also, verbal motivation and online visual feedback were provided.

#### 2.6.1. Maximal Knee Extensor Strength

Maximal isometric strength of the knee extensor muscles was evaluated by use of an isokinetic dynamometer (Kin-Com, Chattecx, USA) [3, 4], as previously described in detail [4]. The trial with the highest moment of force was selected and presented in absolute values (Nm).

#### 2.6.2. Handgrip Strength

Maximal isometric strength of the handgrip muscles was evaluated by use of a custom-build dynamometer setup. Subjects were standing with their arms fully extended gripping around the dynamometer (adjusted to individual finger length). Force data were sampled, analysed, and presented in N.

#### 2.6.3. Plantar Flexor Strength

Maximal isometric muscle strength of the plantar flexor muscles was evaluated by use of a custom-built dynamometer setup (Kistler piezoelectric force transducer [2]). Subjects were seated with their leg fully extended (adjusted individually) and a 5° dorsal flexion in their ankle joint. Force data were sampled, analysed, and presented in N.

#### 2.6.4. Leg Extension Power

Unilateral lower limb muscle power was evaluated using the Nottingham leg extensor power rig [21]. Participants were seated in the power-rig chair (adjusted to individual leg length, 20° knee joint angle at full extension), while pushing the footplate connected to a flywheel as hard and fast as possible. A minimum of 5 trials with visual feedback was performed. The highest trial is presented in absolute values (W).

#### 2.6.5. Voluntary Muscle Activation

Maximal voluntary muscle activation (neural drive) of the knee extensor muscles was evaluated by use of the superimposed twitch technique, as previously described [4]. Voluntary muscle activation was calculated as the ratio between two doublet twitch stimulation amplitudes, that is, the superimposed force response elicited during maximal knee extension expressed relative to the force response measured during the subsequent muscle resting phase. The trial with the highest activation is presented in percentage of maximal muscle activation (%).

### 2.7. Muscle Biopsy Sampling and Immunohistochemistry

Muscle biopsies were collected with a 5 mm Bergström needle from* m. vastus lateralis* under local anaesthetic (1% lidocaine; Amgros, Denmark). First myosin heavy chain isoform one (MHC-I) (1 : 2000, M8421, Sigma-Aldrich) and then MHC-II (1 : 2000, M4276, Sigma-Aldrich) were detected by standard immunohistochemistry (Vector Laboratories, Denmark). Analyses were performed using Axio imager M1 and Axio Vision by Zeiss (Brock & Michelsen, Denmark). The percentage of type I and II fibres, the cross sectional area (CSA), and the percentage of fibres in each of three groups (“Small-sized fibres”: CSA = 0–2999 *μ*m^2^, “normal-size fibres”: CSA = 3000–9900 *μ*m^2^, and “large-sized fibres”: CSA > 10.000 *μ*m^2^) were determined including all fibres from each patient (85–651 fibres/biopsy). We defined “large-sized” fibres as being >10.000 *μ*m^2^, since this is at least twice the size of healthy, age-matched fibres [22].

### 2.8. Data Presentation and Statistical Analysis

Data are reported as mean ± SD unless otherwise stated. Data in Figure 2 are presented as the change during the control period and the training period, respectively. Statistical significance was tested with a paired *t*-test. For the histochemical analysis of CSA, differences between BL, Pre, and Post were tested with repeated measures two-way ANOVA followed by Bonferroni post hoc testing. Asterisks indicate ^*∗*^*p* < 0.05.

## 3. Results

**Figure 1:**
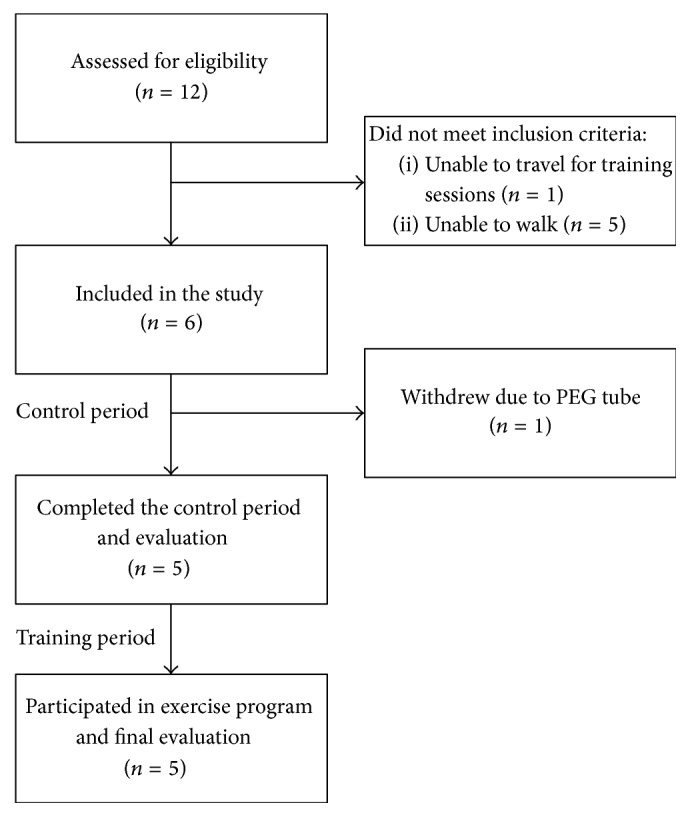
Study flow chart. Participant numbers and withdrawals during the study are listed.

A flow chart describing participant numbers is shown in Figure 1, and baseline characteristics are presented in Table 1. One patient left the study by the end of the control period due to percutaneous endoscopic gastrostomy (PEG) surgery and is thus excluded from all data calculations. Three of the participants completed 85–95% of all planned training sessions, while the remaining two participants completed 50–60% of training sessions due to medical problems not related to the exercise program (intramuscular tumour involving surgery and breathing difficulties due to muscle weakness resulting in a collapse of the back).

### 3.1. The Revised ALS Functional Rating Scale

**Figure 2:**
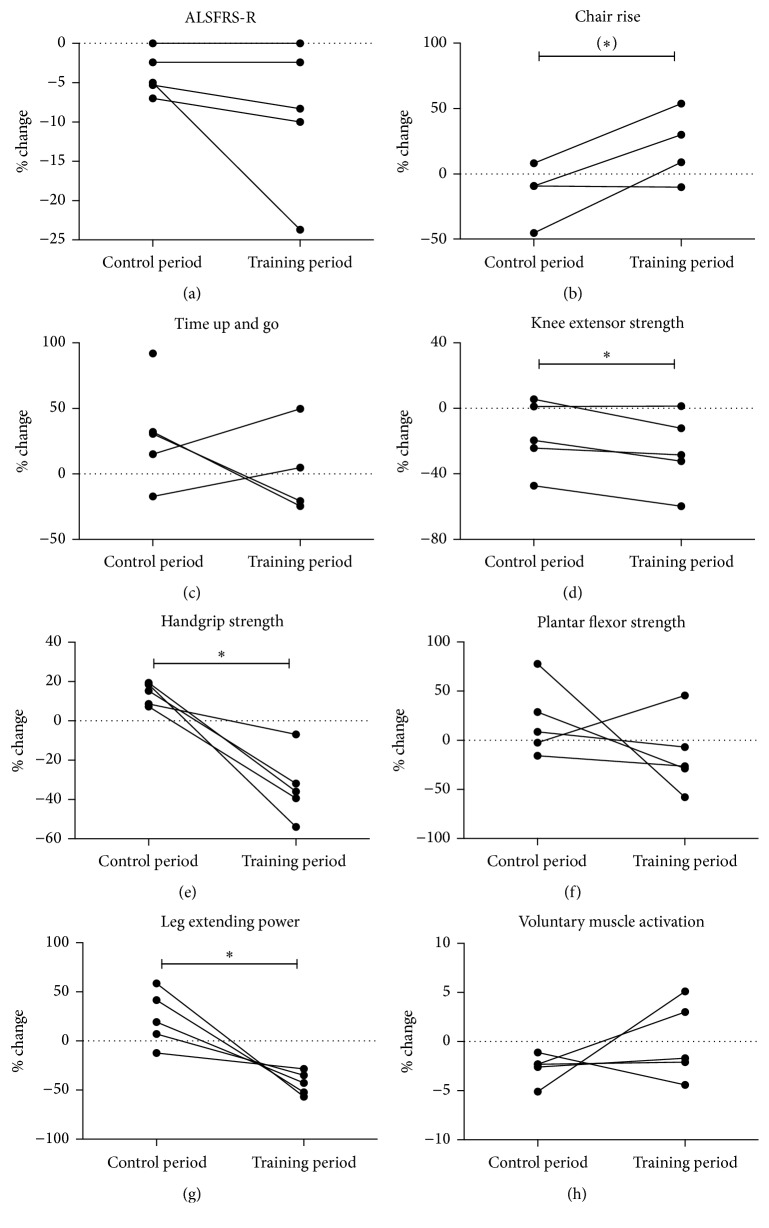
Functional performance and neuromuscular function. (a) Individual change in total scores of the revised ALS functional rating scale (ALSFRS-R). Changes in functional performance in (b) chair rise and (c) timed up and go (TUG) during control and training periods. Note that one participant was unable to perform chair rise at Pre and Post as well as TUG at Post; thus these data are excluded. Change in maximal voluntary muscle strength (MVC) of (d) knee extensors, (e) handgrip, and (f) plantar flexor during the control and training period. (g) Change in explosive leg extension power. (h) Voluntary muscle activation measured by doublet twitch stimulations. Dots represent individual values of change from BL to Pre (control period) and from Pre to Post (training period).

Mean total ALSFRS-R scores were 40.2 ± 2.3 versus 38.6 ± 1.9 versus 35.2 ± 4.3 at BL, Pre, and Post, respectively. ALSFRS-R scores decreased to the same extent during control and training periods in two participants, while they decreased more in training versus control period in three participants (Figure 2(a)).

### 3.2. Functional Measures

The mean number of chair rises was 9.6 ± 2.9 at baseline, 8.4 ± 3.8 at Pre, and 10.4 ± 6.5 at Post. The mean time for completing the TUG was 15.9 ± 5.9 versus 17.9 ± 6.9 versus 17.8 ± 6.8 seconds at BL, Pre, and Post, respectively. Figures 2(b) and 2(c) show the individual % change following control and training periods. Generally, the participants improved their performance in chair rise following the training period (*p* < 0.06), while two individuals improved and two individuals worsened in TUG following the training period.

### 3.3. Neuromuscular Function (Strength and Power)

Neuromuscular function was evaluated by use of well-known tests of strength and power (Table 2). Generally, participants performed differently depending on individual muscle involvement and disease progression. The individual percentage change demonstrates an overall higher decrease in strength during the training period compared to the control period, which was significant (*p* < 0.05) for knee extensor, handgrip, and leg extensor strength (Figures 2(d)–2(g)).

### 3.4. Voluntary Muscle Activation

As an indicator of the neural drive to the knee extensor muscles, voluntary muscle activation was evaluated by use of the superimposed twitch technique. Voluntary muscle activation was 96.1 ± 5.3% versus 93.5 ± 6.5% versus 93.4 ± 4.5% at BL, Pre, and Post, respectively. Four of the five subjects had maximal voluntary muscle activation (>95%) when initiating the study. Yet, two out of five participants increased their voluntary muscle activation during training, and two had smaller decreases compared to control period (Figure 2(h)).

### 3.5. Muscle Fibre Morphology

**Figure 3:**
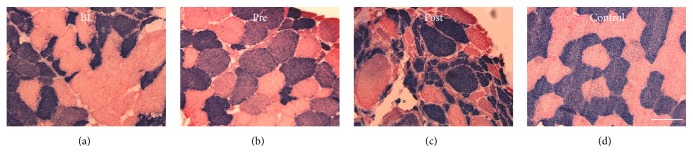
Muscle morphology. (a–d) Representative images of the histochemical staining of type I (blue) and type II fibres (red) from BL, Pre, and Post. A healthy age-matched control is included as a reference. Scale bar = 100 *μ*m.

Muscle fibre type composition (type I versus type II) did not change through the study (Table 3). Muscle fibres ranged from less than 100 *μ*m^2^ to more than 18.000 *μ*m^2^ (Figures 3(a)–3(d)), with the same muscle sample often including both atrophied and hypertrophied fibres. The average CSA did not change between time points (type I fibres: 3480 ± 336 *μ*m^2^ versus 3117 ± 670 *μ*m^2^ versus 3567 ± 922 *μ*m^2^, resp.; type II fibres: 3998 ± 528 *μ*m^2^ versus 3461 ± 1011 *μ*m^2^ versus 3393 ± 1517 *μ*m^2^, resp.). The percentage of small-sized type II fibres (representing atrophied fibres) was larger at Pre and Post compared to BL (*p* < 0.05) (Table 3). Also, the percentage of large fibres was higher at Post compared to BL (*p* < 0.05). Consequently, percentage of normal-sized fibres was smaller at Pre and Post compared to BL.

## 4. Discussion

Since little information can be found in the literature about the neuromuscular effects of resistance training in ALS patients, this study explored the effect of resistance training on neuromuscular function and voluntary muscle activation in this patient group. In addition, the concurrent effect of resistance training on skeletal muscle morphology was evaluated.

The main finding was that resistance training did not attenuate the decline in ALSFRS-R, a finding that was also supported by our results on neuromuscular function (strength and power). However, our observations on functionality, voluntary muscle activation, and CSA indicate that resistance training was able to affect the skeletal muscle.

The present study found a mean decline on the revised ALSFRS of 1.6 points during the control period and of 3.4 points during the training period. A previous study has reported ALSFRS-R to decline 0.92 ± 0.08 points per month [23], which is in line with our data (overall decline: 0.83 ± 0.64 points per month). The same study suggests a change of 20% or greater in the slope of the ALSFRS-R to be clinically meaningful [23], indicating that the resistance training in our study had no attenuating effect; rather it might have had a worsening effect. This observation differs from three previous studies, reporting positive effects on ALSFRS-R scores following resistance training using different regimes [9–11]. In one of these studies the training period was 6 months rather than 12 weeks as in our study. If we had extended the training period accordingly this may have changed the results. However, considering that our patients after 3 months of training showed no neuromuscular functional improvement, it is most unlikely that extending the training period would have benefitted the patients.

Generally we observed an increased loss of muscle strength (knee extensor, handgrip, and plantar flexor) and power during the training versus the control period. Since all participants were familiarized with the different tests, these results were unlikely due to a learning effect. Previous studies using progressive resistance training programs similar to the present have proven effective in improving muscle strength and functionality in frail older individuals [2, 4, 24]. Resistance training interventions in ALS patients reporting positive results have used individualized moderate-intense exercises yet without providing more specific details on the exercises, intensity, progression, and so on [6, 9–11]. These aspects probably explain some of the discrepancy in previous findings compared to the present findings, along with the heterogeneity and size of the ALS study populations. In our small cohort of patients, one of the subjects had a very slowly developing form of ALS. It could be considered that inclusion of this subject may have biased the results; however, if we remove this patient from our analyses, it does not change the outcome; thus all analyses presented here include all 5 subjects. Others aspects such as the interactions between signalling pathways for protein synthesis and protein degradation could also have played a role and would be worth investigating in future studies.

Voluntary muscle activation has been shown to improve in older individuals following resistance training [4], which is in line with our findings of a diminished decrease after the training period versus the control period. The level of voluntary muscle activation was generally very high, making little room for improvements as previously shown [4]. It is the general view that when spinal motor neurons die, the remaining motor neurons become exposed to an increased work demand in order to compensate and produce the same muscle forces required to perform activities of daily living. One subject was diagnosed with ALS 15 years prior to inclusion in the study (i.e., having slowly progressing ALS) and was the one having a low level of voluntary muscle activation at baseline. It is likely that physical inactivity and disuse may have taken part in reducing the voluntary muscle activation level as previously shown in the aging population.

The latter point is of significance in relation to applying resistance training for ALS patients. If a person—with or without ALS—is physically inactive, loss of neuromuscular function will inevitably occur, subsequently leading to a progressive loss of physical capacity. Hence, a combination of disuse- and disease-induced muscle weakness might cause additional detrimental effects in ALS patients.

Muscle disuse and sarcopenia leads to a reduced fibre CSA, while resistance training has the opposite effect eliciting increases in CSA in young and old people [22]. Particularly hypertrophy of type II fibres is seen as a compensatory mechanism and is also seen in overloaded skeletal muscle of patients with neuromuscular diseases such as ALS [25].

The increase in the percentage of “small-sized” type II fibres with time was accompanied by an increase in “large-sized” type II fibres at Post and a concurrent decrease in “normal-sized” fibres. While the hypertrophy of single fibres may have been a result of compensatory MU “overload” during resistance training (i.e., indicating a morphological training effect), it may also be due to disease progression in itself. In any case these changes in fibre size distribution apparently were unable to compensate for the disease-induced progressive loss of motor neurons, which contributed to the continuous decrease in neuromuscular function.

The participants received protein supplements after each training session to ensure proper nutritional status; however issues of tissue oxygenation and potential hypoxia were not investigated in the present study and might have played a role in attenuating the hypertrophic response [26]. Oxygen capacity could be investigated in a future study.

The present study holds some limitations. The most obvious is the small number of patients included in the stury and their heterogeneous disease progression. However as the participants represent an entire population of ALS patients from Funen, Denmark, we may be able to generalise our findings to larger populations of ALS patients. As for the study design, we chose a study design with a “lead-in” control period to take into account the individual disease progression, rather than a case-control design. The ladder would have required a highly effective paring in regard to sex, age, and disease progression between controls and exercisers. We did not find it reasonable to perform a large-scale randomized controlled trial (RCT) before the applied methods had been tested in a small-scale study. Yet, a major strength of the present study is the longitudinal collection of functional and neuromuscular performance data combined with concurrent muscle biopsy sampling, which is unique in ALS patients and it is our hope that the present findings can serve as a background for planning future studies.

## Figures and Tables

**Table 1:** Clinical characteristics of the participants at baseline. Subject 6 left the study by the end of the control period and is thus excluded from all data calculations except this table.

Subject	1	2	3	4	5	6	Mean
Age (years)	68	47	65	69	57	69	62.2 ± 8.2
Sex	Female	Male	Male	Male	Male	Male	N/A
Disease duration (months)	<12	<12	<12	180	<12	<12	N/A
Site of onset	Spinal	Spinal	Bulbar	Spinal	Spinal	Bulbar	N/A
ALSFRS-R	42	40	38	38	43	37	39.7 ± 2.4
Riluzole treatment	−	−	−	−	+	−	N/A
Height (cm)	164	176	178	169	182	171	173 ± 6.0
Weight (kg)	62	74	67	79	85	82	74.8 ± 8.2

**Table 2:** Mean values of strength and power measures.

Evaluation method	*n* = 5	Baseline	Pre	Post
Knee extensor strength	Nm	59.6 ± 31.1	54.6 ± 38.2	46.9 ± 41.1
Hand grip strength	N	199.8 ± 87.5	226.6 ± 95.0	142.6 ± 55.4
Plantar flex	N	59.9 ± 38.5	69.8 ± 44.4	51.9 ± 30.1
Leg power	W	93.0 ± 65.1	101.9 ± 59.0	62.3 ± 44.0

**Table 3:** Muscle fibre morphology.

	Total fibres	Small fibres	Normal fibres	Large fibres
Type I fibres (%)				
Baseline	52.5 ± 6.3	54.1 ± 3.0	43.6 ± 1.4	2.3 ± 0.32
Pre	54.9 ± 9.3	58.2 ± 6.1	39.5 ± 2.9	2.3 ± 0.37
Post	62.7 ± 14.5	56.5 ± 7.6	36.2 ± 2.6	7.4 ± 0.94

Type II fibres (%)				
Baseline	42.7 ± 6.7	44.6 ± 3.6	51.6 ± 1.6	3.8 ± 0.40
Pre	40.3 ± 7.5	58.3 ± 7.1^a^	35.6 ± 2.3^a^	6.0 ± 0.78
Post	35.3 ± 9.0	63.3 ± 9.1^a^	23.9 ± 1.7^a,b^	12.8 ± 2.3^a^

Morphological analysis of fibre type composition (total type I fibres and total type II fibres) and cross-sectional area (CSA) defined as “small-sized fibres” (CSA 0–2999 *μ*m^2^), “normal-size fibres” (CSA 3000–9900 *μ*m^2^), and “large-sized fibres” (CSA > 10.000 *μ*m^2^). a denotes differences from baseline and b denotes difference from pre; *p* < 0.05.
